# Data Archive Challenge: Transitioning Users to New IDs and Data File Format at the Protein Data Bank

**DOI:** 10.1063/4.0000921

**Published:** 2025-10-27

**Authors:** Brian P Hudson, Zukang Feng, Irina Persikova, Yuhe Liang, Ezra Peisach, Jasmine Y Young, wwPDB Team, Stephen K Burley

**Affiliations:** 1 RCSB Protein Data Bank, Rutgers, The State University of New Jersey, Piscataway, NJ, USA; 2 PDBe, EMBL-European Bioinformatics Institute, Hinxton, -, United Kingdom; 3 PDBj, Institute for Protein Research, Osaka, -, Japan; 4 EMDB, EMBL-European Bioinformatics Institute, Hinxton, -, United Kingdom; 5 BMRB, UConn Health, Farmington, CT, USA; 6 PDBc, ShanghaiTech University and National Facility for Protein Science in Shanghai, Shanghai, -, China; 7 RCSB Protein Data Bank, San Diego Supercomputer Center, University of California San Diego, San Diego, CA, USA

## Abstract

The Protein Data Bank (PDB), established in 1971 as the first open-access digital resource in biology, has grown from seven X-ray crystallographic structures to over 233,000 3D structures of biological macromolecules supporting research and education across various scientific fields, including fundamental biology, biomedicine, biotechnology, and energy sciences. The Worldwide Protein Data Bank (wwPDB, wwpdb.org) partnership includes five Full Members and one Associate Member, all committed to making structural biology data Findable, Accessible, Interoperable, and Reusable (FAIR).

Due to the rapid increase in structure depositions, the wwPDB anticipates exhausting the current four-character PDB IDs by 2028. To prepare for this milestone, the PDB is transitioning to extended IDs, formatted as "pdb_" followed by eight alphanumeric characters (e.g., pdb_10021abc). Once four-character PDB IDs are exhausted, new entries will receive only extended IDs and will be available exclusively in the PDBx/mmCIF format, which is flexible, extensible, and can accommodate 3D biostructures of any size and complexity.

PDB depositors, data users, software developers, and journals will all feel the impact of this change. Herein, we present a five-year transition plan aimed at facilitating the adoption of extended PDB IDs and the exclusive use of PDBx/mmCIF format data files. The presentation will include a guide to extended PDB IDs and the PDBx/mmCIF format, along with illustrative examples.

We will also outline our communication strategy for engaging user communities throughout this transition. Resources to support users will include FAQs, training materials, software tools, and a beta PDB archive for users and developers to test extended IDs prior to their full incorporation into the archive. Key updates will be disseminated via presentations, flyers, and news releases. We invite meeting participants to contribute suggestions for promoting this initiative.

RCSB PDB is funded by the National Science Foundation (DBI-2321666), US Department of Energy (DE-SC0019749), National Cancer Institute, National Institute of Allergy and Infectious Diseases, National Institute of General Medical Sciences of the National Institutes of Health (R01GM157729).

